# Postsynaptic Density Protein 95 in the Striosome and Matrix Compartments of the Human Neostriatum

**DOI:** 10.3389/fnana.2015.00154

**Published:** 2015-11-30

**Authors:** Ryoma Morigaki, Satoshi Goto

**Affiliations:** ^1^Department of Neurodegenerative Disorders Research, Institute of Biomedical Sciences, Graduate School of Medical Sciences, Tokushima UniversityTokushima, Japan; ^2^Parkinson’s Disease and Dystonia Research Center, Tokushima University Hospital, Tokushima UniversityTokushima, Japan; ^3^Department of Neurosurgery, Institute of Biomedical Sciences, Graduate School of Medical Sciences, Tokushima UniversityTokushima, Japan

**Keywords:** PSD-95, dopamine D1 receptor, neostriatum, neurodegeneration, striosome, matrix

## Abstract

The human neostriatum consists of two functional subdivisions referred to as the striosome (patch) and matrix compartments. The striosome-matrix dopamine systems play a central role in cortico-thalamo-basal ganglia circuits, and their involvement is thought to underlie the genesis of multiple movement and behavioral disorders, and of drug addiction. Human neuropathology also has shown that striosomes and matrix have differential vulnerability patterns in several striatal neurodegenerative diseases. Postsynaptic density protein 95 (PSD-95), also known as disks large homolog 4, is a major scaffolding protein in the postsynaptic densities of dendritic spines. PSD-95 is now known to negatively regulate not only *N*-methyl-D-aspartate glutamate signaling, but also dopamine D1 signals at sites of postsynaptic transmission. Accordingly, a neuroprotective role for PSD-95 against dopamine D1 receptor (D1R)-mediated neurotoxicity in striatal neurodegeneration also has been suggested. Here, we used a highly sensitive immunohistochemistry technique to show that in the human neostriatum, PSD-95 is differentially concentrated in the striosome and matrix compartments, with a higher density of PSD-95 labeling in the matrix compartment than in the striosomes. This compartment-specific distribution of PSD-95 was strikingly complementary to that of D1R. In addition to the possible involvement of PSD-95-mediated synaptic function in compartment-specific dopamine signals, we suggest that the striosomes might be more susceptible to D1R-mediated neurotoxicity than the matrix compartment. This notion may provide new insight into the compartment-specific vulnerability of MSNs in striatal neurodegeneration.

## Introduction

The human striatum consists of the neostriatum (i.e., the caudate nucleus and putamen) and the nucleus accumbens. The neostriatum is divided into two functional subdivisions referred to as the striosome (patch) and matrix compartments, which are developmentally, anatomically, and biochemically distinct (Graybiel, 1990; Gerfen, 1992). Medium spiny neurons (MSNs) are the major constituent of both the striosome and matrix compartments, and their dendrites and local axon collaterals are largely confined within the same compartment (Walker et al., 1993; Yung et al., 1996; Hanley and Bolam, 1997; Fujiyama et al., 2011). Since the matrix compartment makes up approximately 80% of the volume of the striatum, matrix MSNs forms a major striatal efferent system that projects the direct and indirect pathways (Crittenden and Graybiel, 2011). In addition to their enrichment in the dopamine D1 receptors (D1Rs), striosomal MSNs are unique among striatal cells in sending their GABAergic projections directly or indirectly to the substantia nigra pars compacta (SNc), which contains dopamine-producing cells that project back to both the striosome and matrix compartments (Gerfen, 1984; Jimenez-Castellanos and Graybiel, 1989; Tokuno et al., 2002; Fujiyama et al., 2011; Watabe-Uchida et al., 2012). Accordingly, striosomal MSNs could be in a position to exert global control over dopamine signals in the neostriatum by inhibiting the activity of dopamine-producing cells in the SNc. The striosome-matrix dopamine systems play a central role in cortico-thalamo-basal ganglia circuits (Graybiel, 2008; Amemori et al., 2011), and their involvement is thought to underlie the genesis of multiple movement and behavioral disorders, and of drug addiction (for review see, Graybiel, 2008; Goto et al., 2010; Crittenden and Graybiel, 2011). Moreover, human neuropathology has shown that striosomes and matrix have differential vulnerability patterns in several striatal neurodegenerative diseases, such as Huntington’s disease (HD; OMIM 143100) (Crittenden and Graybiel, 2011).

Postsynaptic density protein 95 (PSD-95), also known as disks large homolog 4 (DLG4), is the best characterized of the synaptic PDZ proteins (Kim and Sheng, 2004; van Zundert et al., 2004). PSD-95 is identified as a member of the membrane-associated family of guanylate kinases and as a major scaffolding protein in the PSD of dendritic synapses (Kim and Sheng, 2004; van Zundert et al., 2004). PSD-95 interacts not only with the *N*-methyl-D-aspartate (NMDA) glutamate receptors but also the D1Rs at sites of synaptic transmission (Fiorentini et al., 2003; Zhang et al., 2007; Sun et al., 2009; Ha et al., 2012). Evidence also has suggested that in striatal neurons, PSD-95 could act as a negative regulator for the synaptic activity mediated by D1Rs and NMDARs (Zhang et al., 2007, 2009; Yao et al., 2008). Maladaptive functioning of PSD-95 has been associated with a variety of pathological brain conditions (Migaud et al., 1998; Sattler et al., 1999; Gardoni et al., 2002; Yao et al., 2004; Porras et al., 2012; Parsons et al., 2014).

In this study, we used a highly sensitive immunohistochemistry technique (Goto et al., 2015) to identify PSD-95 and D1R in formalin-fixed paraffin-embedded tissue from autopsied human brains. Our results from the human neostriatum showed that the striosomes are enriched with D1R but show a paucity of PSD-95 compared with the matrix. Given the possible involvement of PSD-95-mediated synaptic function in compartment-specific dopamine signals, we suggest that the complementary distribution of PSD-95 and D1R in the striosome and matrix compartments might underlie the compartment-specific vulnerability of MSNs in striatal neurodegenerative disorders such as HD.

## Materials and Methods

### Western Blot Analysis

Male C57BL/6 mice (Nihon SLC Co., Shizuoka, Japan), 8–10 weeks of age, were used. All procedures involving experimental mice were approved by the Ethical Review Committee of the University of Tokushima. The mice were sacrificed by cervical dislocation and transcardially perfused with ice cold PBS. The heads of the mice were immediately immersed in liquid nitrogen for 5 s. The dissected striatal tissue samples were homogenized in a homogenizing buffer containing 50 mM Tris-HCl, pH 7.5, 0.5 M NaCl, 1% CHAPSO, 1 mM MgCl_2_, 1 mM dithiothreitol, and a protease-inhibitor cocktail (Pierce Biotechnology, Inc., Rockford, IL, USA). After removal of insoluble materials by centrifugation at 12,000 rpm for 10 min, the homogenates were solubilized in Laemmli sample buffer. Ten micrograms of protein from each sample were separated on 10% SDS-PAGE gels. Separated proteins were electrophoretically transferred to polyvinylidene difluoride (PVDF) membranes (ATTO, Tokyo, Japan) at 70 V for 1.5 h using a wet blotting system. The PVDF membranes were incubated for 1 h at room temperature with Tris-buffered saline containing 0.1% Tween 20 (TBST) and 0.5% skim milk, followed by overnight incubation at 4°C with a rabbit polyclonal antibody against PSD-95 (1:5,000; Cell Signaling Technology, Danvers, MA, USA) in TBST containing 0.5% skim milk. After several rinses in TBST, the membranes were incubated with a horseradish peroxidase-conjugated secondary antibody in TBST for 1 h. Immunoreactive bands were visualized by enhanced chemiluminescent autoradiography (ECL plus kit; GE Healthcare, Buckingham, UK).

### Immunohistochemical Detection of PSD-95 in Mouse Brains

Mice (Nihon SLC Co.; *n* = 5) were injected intraperitoneally with a lethal dose of pentobarbital (Sigma-Aldrich, St. Louis, MO, USA), and were then transcardially perfused with 0.01 M phosphate-buffered saline (PBS) at pH 7.2, followed by cold 4% paraformaldehyde in 0.1 M phosphate buffer (PB) at pH 7.2. The brains were removed, post-fixed overnight in the same fixative at 4°C, and stored in a 10–30% sucrose gradient in 0.1 M PB at 4°C for cryoprotection. Sections were cut on a cryostat at 16-μm thickness, and stored in PBS containing 0.05% NaN_3_ until use. Immunostaining was performed on free-floating sections using the tyramide signal amplification (TSA) method, according to our previous report (Okita et al., 2012). After blocking endogenous peroxidase activity, the sections were incubated in PBS containing 3% BSA for 60 min. They were then incubated in PBS-BSA with anti-PSD-95 antibody (1:10,000; Cell Signaling) for 18 h. The bound antibody was detected using the Histofine Simple Stain Kit (Nichirei, Tokyo, Japan) and the TSA-system with Cyanine3 (Perkin Elmer, Shelton, CT, USA).

### Autopsied Human Brain and Tissue Preparation for Immunohistochemistry

All procedures involving postmortem human brain tissue were approved by the Ethical Review Committee of the Tokushima University.

Human brains were obtained at autopsy from neurologically normal individuals (*n* = 5; mean age ± SEM, 59 ± 8 years). Brain tissue was routinely fixed in 10% neutral buffered formalin for about 3 weeks, and then embedded in paraffin. Later, 4-mm-thick sections were prepared on a microtome and mounted onto MAS-coated glass slides (Matsunami Glass, Osaka, Japan). After routine deparaffinization, rehydration, and blocking of endogenous peroxidase activity with 1% H_2_O_2_ in water for 5 min, all sections were immersed in 0.01 M sodium citrate buffer (pH 6.0) and placed in a 700-W microwave oven at maximum power for 15 min. After several rinses in PBS, endogenous avidin and biotin activity was blocked using the Avidin/Biotin Blocking Kit (Vector, Burlingame, CA, USA). Following several rinses in PBS, sections were further blocked in PBS containing 3% BSA for 60 min. All procedures were carried out at room temperature. Summary of the antibodies used in this study is shown in **Table 1**.

**Table 1:** Antibodies used for immunohistochemistry in the human brain tissues.

Antibody to:	Immunogen:	Source	Dilution	
			IHC with DAB	IHC with fluorescence
Postsynaptic density protein 95 (PSD-95)	Synthetic peptide corresponding to residues of human PSD-95	Cell Signaling Technology (Danvers, MA, USA); Catalog No. #2507 Rabbit polyclonal antibody	1:4,000	1:2,000
Calbindin-D28K	Synthetic peptide for the C-terminus of calbindin D28K of human origin	Santa Cruz Biotechnology (Santa Cruz, CA, USA); Catalog No. MAB9627 Goat polyclonal antibody	1:10,000	1:5,000
Dopamine-and cAMP-regulated phosphoprotein, Mr 32 kDa (DARPP-32)	Synthetic peptide for the residues around Thr34 of human DARPP-32	Cell Signaling Technology (Danvers, MA, USA); Catalog No. #2302 Rabbit polyclonal antibody		1:2,000
Dopamine D1 receptor (D1R)	Recombinant fusion protein containing the C-terminal 97 amino acid of human D1R	Sigma-Aldrich (St. Louis, MO, USA); Catalog No. D2944 Rat monoclonal antibody		1:100,000

*IHC, Immunohistochemistry; DAB, 3,3′-diaminobenzidine*.

### Immunohistochemical Detection of a Single Antigen in Human Brain Tissue

The sections were incubated with a rabbit polyclonal antibody against PSD-95 (1:5,000; Cell Signaling) or a goat polyclonal antibody against Calbindin-D28K (1:10,000; Santa Cruz Biotechnology, Santa Cruz, CA, USA) for 18 h in PBS containing 3% BSA. After several rinses in PBS, the sections were incubated with the polymer-staining reagent (Histofine Simple Stain Kit; Nichirei) for 30 min. After several rinses in PBS, they were processed for TSA using the TSA Biotin System (Perkin Elmer). Sections were then incubated in the biotinyl tyramide amplification reagent. A working solution was prepared by diluting the Biotinyl Tyramide Stock Solution (Perkin Elmer) 1:50 using 1× Plus Amplification Diluent (Perkin Elmer) for 30 min. After several rinses in PBS, the sections were incubated for 30 min with the avidin-biotin-peroxidase complex (ABC) reagent from a Vectastain Elite ABC kit (Vector). The bound peroxidase was visualized by incubating the sections with a solution containing 0.05% 3,3′-diaminobenzidine (DAB; Merck, Darmstadt, Germany) and 0.01% H_2_O_2_ in 0.05 M Tris-HCl (pH 7.4) for 10 min. The immunostained sections were dehydrated and cover-slipped with Malinol (Muto Pure Chemicals, Tokyo, Japan).

### Immunohistochemical Detection of Dual Antigens in Human Brain Tissue

For dual antigen detection, sections were first incubated in PBS containing 3% BSA and a goat polyclonal antibody against Calbindin-D28K (1:5,000; Santa Cruz), a rabbit polyclonal antibody against dopamine-and cAMP-regulated phosphoprotein, Mr 32 kDa (DARPP-32) (1:2,000, Cell Signaling) or a rat monoclonal antibody against D1R (1:100,000; Sigma–Aldrich) for 18 h. The bound antibody was detected using the Histofine Simple Stain Kit (Nichirei) and the TSA-system with Cyanine3 (Perkin Elmer). To remove bound antibody, the immunostained sections were incubated in 0.1 M glycine-HCl (pH 2.2) for 30 min. After several rinses in PBS, the sections were then incubated for 18 h in PBS containing 3% BSA and anti-PSD-95 antibody (1:2,000; Cell Signaling). The bound antibodies were detected using the Histofine Simple Stain Kit (Nichirei) and the TSA-system with Fluorescein (Perkin Elmer). After several rinses in PBS, the sections were cover-slipped with PBS containing 10% glycerol.

### Digital Images and Densitometry

Macroscopic images were captured using an Epson ES-2200 color image scanner (SEIKO EPSON Co., Nagano, Japan) using the 24-bit color mode. Microscopic images stained with DAB were captured using an Olympus BX51 microscope (Olympus, Tokyo, Japan) equipped with a digital camera DP40 (Olympus). The digital images were imported into Adobe Photoshop CS4 and digitally processed for the minimal adjustment of contrast, brightness, and color balance.

The somatic density of PSD-95 labeling in the striatal neurons was estimated, as in our previous report (Okita et al., 2012). High-power photomicrographs of labeled neurons were obtained using a 100× oil-immersion objective, and they were digitally changed to the non-colored images in a gray scale. We measured the optical density of PSD-95 labeling in the soma of striatal neurons (*n* = 20) in each human striatal section (*n* = 5). The mean somatic density of PSD-95 labeling was then calculated in each. The optical densities of PSD-95- or D1R-immunoreactive products in the striosome and matrix subfields were also measured as gray levels on non-colored digital images at a low-power magnification, as in our previous report (Sato et al., 2008). For each human striatum (*n* = 5), measurements were made in 5 striatal subfields from five sections.

### Statistical Analysis

All quantitative data were expressed as means ± SEM values. The Student’s *t*-test (two-tailed, paired) was used for two group comparisons. *P*-values less than 0.05 were considered statistically significant.

## Results

### Immunochemical Detection of PSD-95 in Mouse Brains

**FIGURE 1:**
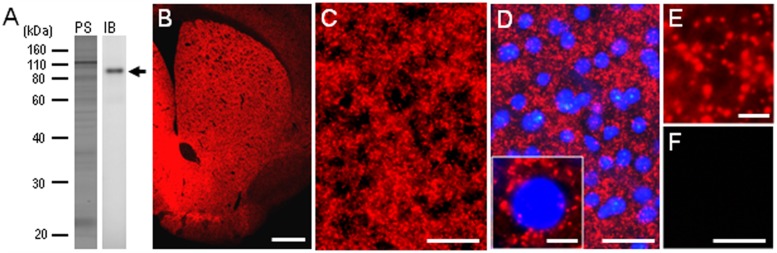
**Identification of postsynaptic density protein 95 (PSD-95) in the mouse striatum. (A)** Western blot assay. Crude homogenates of protein (10 μg) from the mouse striatum were separated on a 10% gel by SDS-PAGE and then immunoblotted using anti-PSD-95 antibody. Note that an immunostained protein band (arrow) was selectively detected, with an approximate molecular mass corresponding to the predicted size of native PSD-95 protein. PS, protein staining; IB, immunoblot. **(B)** Photomicrograph of a striatal section stained for PSD-95. **(C–E)** Photomicrographs of the dorsal striatum stained for PSD-95 in the absence **(C,E)** and presence **(D)** of DAPI (4′,6-diamidino-2-phenylindole)-staining. Tiny dots immunoreactive for PSD-95 (*inset* in **D,E**) are numerously found in the dorsal striatum. **(F)** Photomicrograph of the dorsal striatum processed using the immunostaining protocol without anti-PSD-95 antibody. Scale bars: **(B)** 1 mm, **(C,D,F)** 50 μm, (*inset* in **D**) 5 μm, **(E)** 2.5 μm.

To confirm the specificity of the anti-PSD-95 antibody used here, we first carried out a western-blot analysis of the mouse brains. A protein band with an approximate molecular mass corresponding to the predicted size of native PSD-95 protein was selectively detected on the immunoblots of mouse striatal extracts (**Figure 1A**). The specificity of staining was also determined on frozen sections from mouse brains with or without anti-PSD-95 antibody (**Figures 1B–F**). Strong immunoreactivity for PSD-95 was found in the striatum (**Figure 1B**), where numerous tiny immunoreactive dots were densely distributed (**Figures 1C–E**). According to the previous reports (Kim and Sheng, 2004; van Zundert et al., 2004), we suppose that the vast majority of them were localized in the PSDs of dendritic spines of striatal neurons. No immunoreactivity for PSD-95 was found in striatal sections processed using the immunostaining protocol without the anti-PSD-95 antibody (**Figure 1F**). Notably, no apparent compartmental localization of PSD-95 labeling in the mouse striatum could be detected (**Figure 1B**). A knockout control for specificity of reactivity in immunohistological experiments was not done.

### Immunohistochemical Detection of PSD-95 in the Human Neostriatum

**FIGURE 2:**
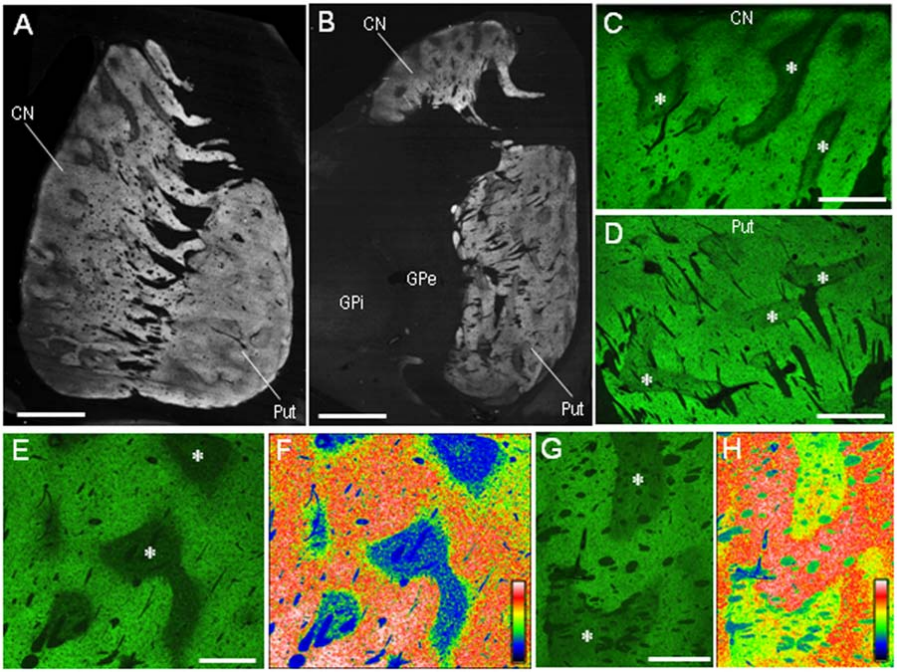
**Non-homogeneous distribution of PSD-95 in the human neostriatum. (A,B)** Dark-field images of the striatum **(A)** and lenticular nucleus **(B)** stained for PSD-95 with DAB. **(C,D)** Photomicrographs of the caudate nucleus **(C)** and putamen **(D)** processed for immunofluorescence staining with anti-PSD-95 antibody. **(E,F)** Displayed are the caudate nucleus subfield stained for PSD-95 **(E)**, and its graded color-converted image **(F)**, in which labeling intensity is indicated in a standard pseudocolor scale from blue (lowest level) through green, yellow, red, and white (highest level). **(G,H)** Displayed are the putamen subfield stained for PSD-95 **(G)**, and its graded color-converted image **(H)**, in which labeling intensity is indicated in a standard pseudocolor scale from blue (lowest level) through green, yellow, red, and white (highest level). Asterisks indicate examples of the striatal subfields with sparse PSD-95 immunoreactivity. CN, caudate nucleus; Put, putamen; GPe, globus pallidus externa; GPi; globus pallidus interna. Scale bars: **(A,B)** 5 mm, **(C,D)** 2.5 mm, **(E–H)** 1.5 mm.

Our highly sensitive immunohistochemical technique allowed us to detect PSD-95 immunoreactivity in formalin-fixed paraffin-embedded human autopsy tissue. Strong PSD-95 labeling was found in the striatum, consisting of the caudate nucleus, putamen, and nucleus accumbens. Notably, in macroscopic images of the rostral (**Figure 2A**) and caudal (**Figure 2B**) parts of the striatum, there was a non-homogeneous distribution of PSD-95 labeling in both the caudate nucleus and putamen. Microscopic images with low-powered magnification also showed the compartmental distribution of PSD-95 labeling in the caudate nucleus (**Figure 2C**) and putamen (**Figure 2D**), and this was more evident in the caudate nucleus (**Figures 2E,F**) than in the putamen (**Figures 2G,H**). No PSD-95 labeling was identified in striatal sections processed using the immunostaining protocol without the anti-PSD-95 antibody.

**FIGURE 3:**
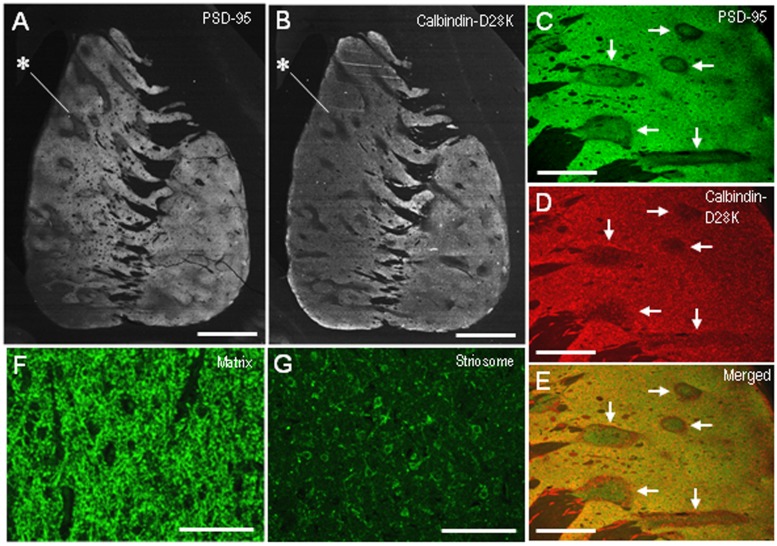
**Postsynaptic density protein 95 immunostaining identifies striatal compartments in the human neostriatum. (A,B)** Serial-section analysis of dark-field images of the striatum stained for PSD-95 **(A)** and Calbindin-D28K **(B)**, a marker for the matrix compartment. Note that the compartmentalization of PSD-95 is almost identical to that of Calbindin-D28K. Examples of striosomes are indicated by asterisks. **(C–E)** Photomicrographs of the striatal area double-stained for PSD-95 **(C)** and Calbindin-D28K **(D)**, with a merged image **(E)**. Corresponding striosomes are indicated by arrows. **(F,G)** Photomicrographs of the matrix **(F)** and striosome **(G)** subfields stained for PSD-95. Scale bars: **(A,B)** 5 mm, **(C–E)** 2 mm, **(F,G)** 100 μm.

**FIGURE 4:**
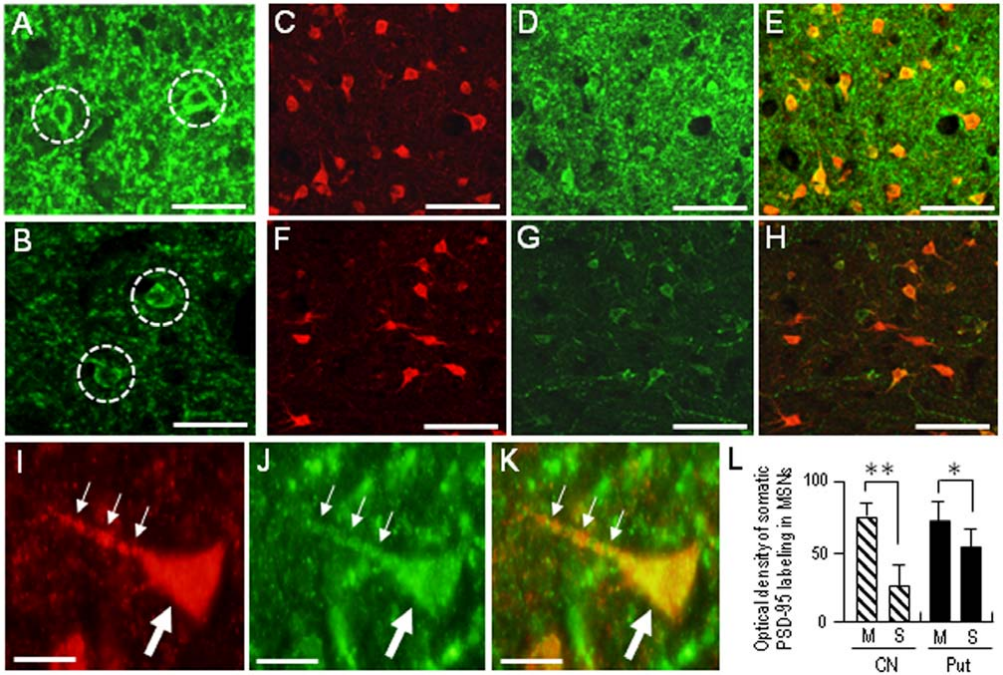
**Somatic labeling for PSD-95 in the medium spiny neurons (MSNs) in the human neostriatum. (A,B)** Photomicrographs of the matrix **(A)** and striosome **(B)** subfields in the striatal section stained for PSD-95. Neurons with somatic labeling for PSD-95 are indicated by dashed open circles. Note that somatic density of PSD-95 labeling in matrix cells is higher than that in striosomal cells. **(C–E)** Photomicrographs of the matrix area double-stained for DARPP-32, a marker protein for striatal MSNs **(C)** and PSD-95 **(D)**, with a merged image **(E)**. **(F–H)** Photomicrographs of the striosome area double-stained for DARPP-32 **(F)** and PSD-95 **(G)**, with a merged image **(H)**. **(I–K)** Photomicrographs of an MSN in the matrix area double-stained for DARPP-32 **(I)** and PSD-95 **(J)**, with a merged image **(K)**. The proximal dendrite and soma of the labeled cell are indicated by small and large arrows, respectively. **(L)** Measurements of the optical densities of somatic PSD-95 labeling of striosomal (S) MSNs and matrix (M) MSNs in the caudate nucleus (CN) and putamen (Put). Data are mean ± SEM (bars) values (*n* = 100). ^∗∗^*p* = 0.01, ^∗^*p* = 0.05, Matrix (M) MSNs vs. Striosomal (S) MSNs. Scale bars: **(A,B)** 20 μm, **(C–H)** 50 μm, **(I–K)** 10 μm.

Compared with the striosomes, the matrix compartment was more strongly stained for PSD-95, as determined using serial sections stained for PSD-95 (**Figure 3A**) and Calbindin-D28K (**Figure 3B**), a protein enriched in the matrix of the human striatum (Ito et al., 1992). Double immunofluorescence staining also showed that PSD-95 immunoreactivity was sparse in striosomes that exhibited low calbindin labeling (**Figures 3C–E**). At higher-powered magnification, PSD-95-immunoreactive dots were found abundantly in the matrix (**Figure 3F**), but less so in the striosomes (**Figure 3G**). Thus, PSD-95 was differentially concentrated in the striosome-matrix systems of the human neostriatum, with higher density of PSD-95 in the matrix relative to the striosomes. In addition, PSD-95 appeared as not only a dendritic but also a somatic protein in striosomal and matrix MSNs (**Figure 4**), as determined using sections double-stained for PSD-95 and DARPP-32, a marker of MSNs (Langley et al., 1997). In both the caudate nucleus and putamen, the mean somatic density of PSD-95 labeling in striosomal MSNs was significantly lower than that in matrix MSNs (**Figure 4L**). This finding suggests that PSD-95 might be abundantly expressed in the matrix MSNs, whereas low levels of PSD-95 expression were observed in the striosomal MSNs.

### Complementary Localization of D1R and PSD-95 in the Human Neostriatum

**FIGURE 5:**
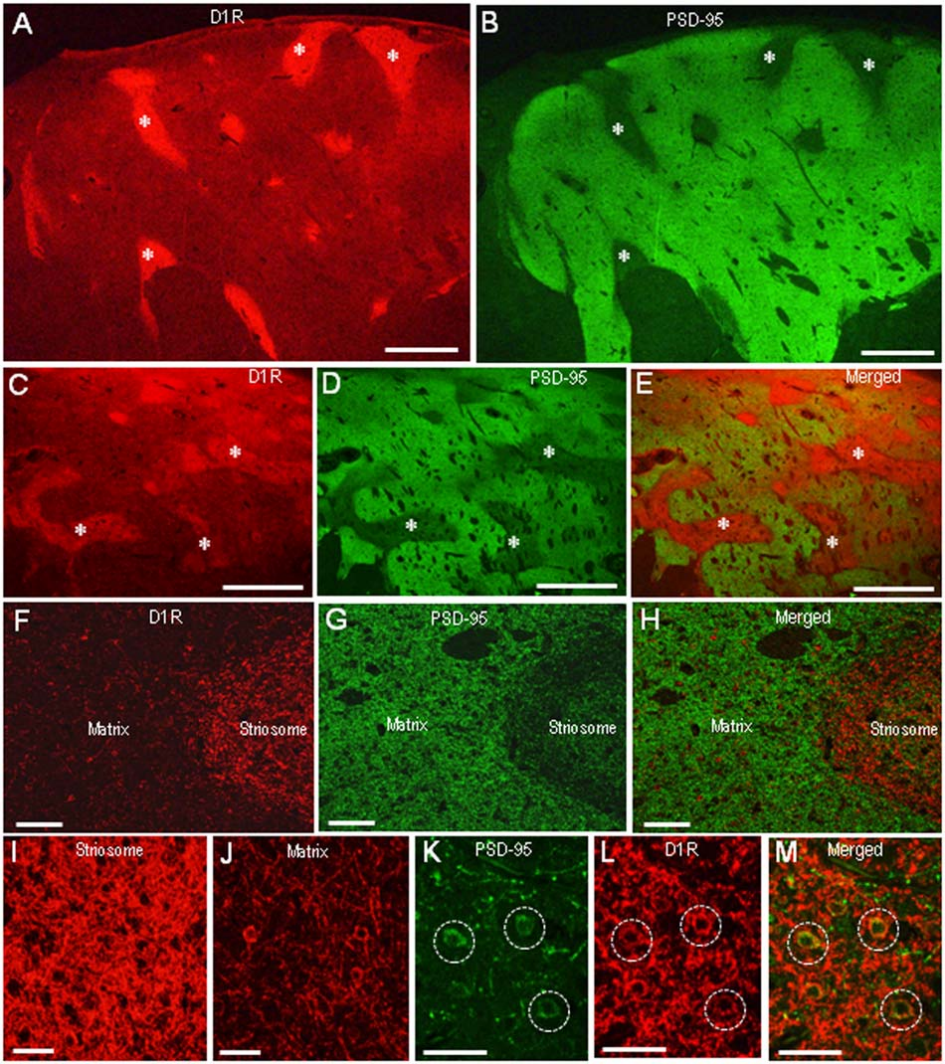
**Complementary distribution of dopamine D1 receptor (D1R) and PSD-95 in striatal compartments of the human neostriatum. (A,B)** Photomicrographs of the caudate nucleus double-stained for D1R **(A)** and PSD-95 **(B)**. Examples of striosomes are indicated by asterisks. **(C–E)** Photomicrographs of the putamen double-stained for D1R **(C)** and PSD-95 **(D)**, with a merged image **(E)**. Examples of the corresponding striosomes are indicated by asterisks. **(F–H)** Photomicrographs showing a border area between the striosome and matrix compartment in the striatal section double-stained for D1R **(F)** and PSD-95 **(G)**, with a merged image **(H)**. **(I,J)** Photomicrographs of the striosome **(I)** and matrix **(J)** areas stained for D1R. **(K–M)** Photomicrographs of the striosomal area double-stained for D1R **(K)** and PSD-95 **(L)**, with a merged image **(M)**. Dashed open circles indicate examples of medium-sized cells possessing both D1R and PSD-95 labeling in their soma. Scale bars: **(A–E)** 2 mm, **(F–H)** 200 μm, **(I–M)** 50 μm.

In agreement with the previous reports (Besson et al., 1988; Levey et al., 1993), we found a compartmental distribution for D1R immunoreactivity in the human neostriatum, with higher labeling density in the striosomes than in the matrix. As determined by double immunofluorescence staining, D1R labeling was strikingly complementary to that of PSD-95 in both the caudate nucleus (**Figures 5A,B**) and putamen (**Figures 5C–E**). At higher-power magnification, the margins of the PSD-95-poor zones appeared to closely correspond with the outer margins of the D1R-rich zones (**Figures 5F–H**). At higher-powered magnification, D1R-immunoreactive products were found abundantly in the striosomes (**Figure 5I**), but less so in the matrix (**Figure 5J**). Striosomal MSNs possessing both D1R and PSD-95 labeling in their soma are shown in **Figures 5K–M**.

**FIGURE 6:**
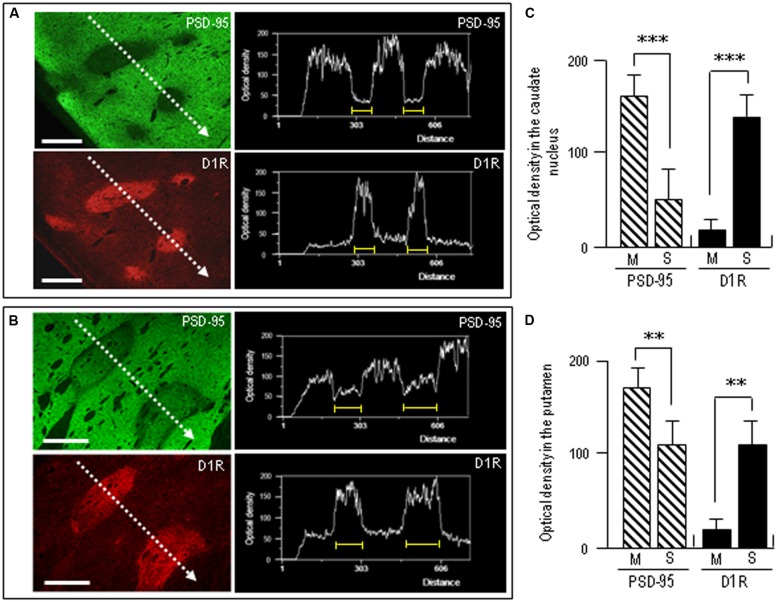
**Densitometric analysis on the compartmental distribution of PSD-95 and dopamine D1R in the human neostriatum. (A,B)** Line scanning densities of two examples of neostriatal areas double-stained for PSD-95 (green) and D1R (red). The striosome in each image is indicated by a yellow bar. Note that striosomal areas with sparse PSD-95 labeling correspond to those enriched in D1R labeling. Scale bars: 1 mm. **(C,D)** Measurements of the optical densities of PSD-95- and D1R-immunoreactive products in the striosome (S) and matrix (M) compartments in the caudate nucleus **(C)** and putamen **(D)**. Data are mean ± SEM (bars) values (*n* = 25). ^∗∗∗^*P* = 0.005, M vs. S; ^∗∗^*P* = 0.01, M vs. S.

To confirm complementary distribution of PSD-95 and D1R, we next carried out a line scanning analysis of the staining density of the neostriatal areas double-stained for PSD-95 and D1R (**Figures 6A,B**). The results showed that striosomal areas poor in PSD-95 labeling were perfectly matched with those enriched in D1R labeling (**Figures 6A,B**). Optical density measurements in the caudate nucleus (**Figure 6C**) and putamen (**Figure 6D**) revealed that PSD-95 labeling in the striosomes was significantly lower than that in the matrix, while D1R labeling in the striosomes was significantly higher than that in the matrix. Thus, in contrast with that in the matrix compartment, the striosomes were enriched in D1R but showed a paucity of PSD-95.

## Discussion

In this study, we documented immunohistochemical evidence showing that the concentrations of PSD-95 and D1R were complementary in the striosome-matrix systems of both the caudate nucleus and putamen in human autopsied brains. A higher density of PSD-95 labeling was found in the matrix relative to the striosomes, while D1R labeling was greater in the striosomes than in the matrix. Since PSD-95 regulates D1R trafficking and sensitization, and restrains dopamine D1 activity in dendritic spines, our results indicate that the striosomes might be more susceptible to increased levels of extracellular dopamine than the matrix, owing to the relative content of not only D1R but also PSD-95. This notion may be implicated in physiological and pathological conditions that are associated with an imbalance in dopamine D1 signaling between the striosome and matrix (for a review, see Crittenden and Graybiel, 2011).

Of particular interest is the possible implication of PSD-95 in the genesis of neuropathology in HD, a major major representative of striatal neurodegenerative disorders (Albin and Tagle, 1995). Striatal pathology in HD is characterized by primary and progressive degeneration of MSNs, with relative sparing of local-circuit interneurons (Cicchetti et al., 2000). Till now, it has been postulated that in HD, the loss of striatal neurons might be caused by excitotoxicity resulting from over activation of postsynaptic NMDARs (Choi, 1988; Zeron et al., 2002; Levine et al., 2004; Fan and Raymond, 2007) and D1Rs (Cyr et al., 2003, 2006; Bozzi and Borrelli, 2006). In accordance with previous evidence indicating that PSD-95 could limit dendritic D1R activity and negatively regulate the D1R and NMDAR interplay that leads to excitotoxicity (Yao et al., 2008; Zhang et al., 2009), Zhang et al. (2014) showed that PSD-95 could exert a neuroprotective effect against the excitotoxic degeneration of striatal MSNs by acting as a molecular brake that dampens postsynaptic activity mediated by dopamine and glutamate signals. Our present finding showed that in contrast with the matrix, the striosomes are enriched in D1R and have a paucity of PSD-95. Taken together, we hypothesize that in HD, the striosomal MSNs might be more susceptible to D1R-mediated excitotoxicity than the matrix MSNs. Indeed, a predominant loss of striatal MSNs in the striosome compartment has been shown in subsets of HD patients (Morton et al., 1993; Hedreen and Folstein, 1995; Tippett et al., 2007) and in a rodent model of HD (Lawhorn et al., 2008).

Our hypothesis may also be relevant to the striatal pathology seen in other disorders such as X-linked dystonia-parkinsonism (XDP/*DYT3*, OMIM314250; Goto et al., 2005, 2013), in which a preferential loss has been documented in the striosome with relative sparing of the matrix compartment. Similar to the findings in HD, XDP (Goto et al., 2005, 2013) also show a preferential loss of MSNs while cholinergic interneurons are spared; this cell-type-specific loss of neurons is a hallmark of striatal excitotoxic lesions (Calabresi et al., 1998, 2000). Dopamine-mediated neurotoxicity may also be involved in the genesis and progression of striatal pathology in XDP (Goto et al., 2005, 2013; Herzfeld et al., 2013). In addition, Herzfeld et al. (2013) transfected human neuroblastoma cells with DSC3, a disease-specific sequence change within the TAF1/DYT3 multiple transcript system, and reported that this exerted a dramatic effect on overall gene expression including multiple genes involved in dopamine metabolism, with a significant decrease in DLG4 (PSD-95) expression. This suggests that a loss of PSD-95 may be involved in the pathogenesis of XDP. In conclusion, our present findings suggest the possible involvement of PSD-95-mediated synaptic function in compartment-specific dopamine signals. This notion also may provide new insight into the compartment-specific vulnerability of MSNs in striatal neurodegenerative diseases.

## Author Contributions

RM: the acquisition, analysis, and interpretation of data for the work; SG: the conception and design of the work; and the acquisition, analysis, and interpretation of data for the work.

## Conflict of Interest Statement

The authors declare that the research was conducted in the absence of any commercial or financial relationships that could be construed as a potential conflict of interest.
